# AntiviralDB: an expert-curated database of antiviral agents against human infectious diseases

**DOI:** 10.1128/mbio.02013-25

**Published:** 2025-09-11

**Authors:** Jie Huang, Qixiang Song, Pan Zhang, Lei Deng, Feng Gao, Yelin Deng, Ewelina Krol, Daniel Růžek, Ricardo Khouri, Erik De Clercq, Guangdi Li

**Affiliations:** 1 Hunan Provincial Key Laboratory of Clinical Epidemiology, Xiangya School of Public Health, Central South University 506614 , Changsha, China; 2 Infection Control Center, Xiangya Hospital of Central South University, Changsha, China; 3 School of Computer Science and Engineering, Central South University 506611 , Changsha, China; 4 Shandong Haoran Information Technology Co., Ltd, Jinan, China; 5 Department of Civil and Environmental Engineering, Soochow University 12582, Suzhou, China; 6 Department of Recombinant Vaccines, Intercollegiate Faculty of Biotechnology, University of Gdansk and Medical University of Gdansk, Gdansk, Poland; 7 Department of Experimental Biology, Faculty of Science, Masaryk University 37748, Brno, Czech Republic; 8 Institute of Parasitology, Biology Centre of the Czech Academy of Sciences 446341 , Ceske Budejovice, Czech Republic; 9 Laboratory of Emerging Viral Diseases, Veterinary Research Institute 48357, Brno, Czech Republic; 10 Laboratory of Precision Medicine and Public Health, Gonçalo Moniz Institute, Oswaldo Cruz Foundation 42509 , Salvador, Brazil; 11 Department of Microbiology, Immunology and Transplantation, Rega Institute for Medical Research 54515 , KU Leuven, Belgium; Centro Nacional de Biotecnologia, Madrid, Spain

**Keywords:** antiviral agents, antiviral targets, clinical guidelines, protocols

## Abstract

Viral infectious diseases have caused millions of deaths worldwide. Antiviral agents are critical for controlling these infections; however, an open-access database dedicated specifically to antiviral agents remains unavailable. Here, we present AntiviralDB (https://www.antiviraldb.com/), an expert-curated resource that compiles both approved and experimental antiviral agents with laboratory-confirmed *in vitro* activity against a broad spectrum of human viruses. These include the human immunodeficiency virus, coronaviruses, hepatitis viruses, influenza virus, respiratory syncytial virus, herpes simplex virus, varicella-zoster virus, human cytomegalovirus, human papillomavirus, dengue virus, Zika virus, Ebola virus, mpox virus, norovirus, chikungunya virus, and 16 other common or life-threatening pathogens. Each antiviral agent in the database is annotated with key information, including its molecular target, *in vitro* antiviral activity (IC_50_, EC_50_, and CC_50_ across specific viral strains and cell lines), mechanism of action, and relevant pharmacokinetic and pharmacodynamic parameters. AntiviralDB also provides clinical efficacy and safety data derived from randomized clinical trials. Unlike existing drug databases, AntiviralDB offers two distinctive features: (i) standardized laboratory protocols for antiviral drug screening under appropriate biosafety conditions and (ii) clinical guidelines for the therapeutic use of antiviral agents against viral infections. By serving as a comprehensive repository of antiviral agents and their clinical applications, AntiviralDB aims to advance antiviral drug discovery and support the effective clinical management of viral infectious diseases.

**IMPORTANCE:**

Over the past decade, viral infectious diseases have caused significant morbidity and mortality worldwide, underscoring the urgent need for effective antiviral therapies. However, there is a lack of an open-access database that consolidates detailed information on antiviral agents, clinical guidelines, and laboratory protocols. To address this need, AntiviralDB was developed to integrate extensive data on antiviral agents—including clinical efficacy, safety profiles, pharmacokinetic parameters, and *in vitro* and *in vivo* activities—together with standardized experimental protocols and clinical treatment guidelines. This resource empowers researchers and clinicians to (i) identify promising antiviral candidates for controlling infectious diseases, (ii) accelerate the discovery and development of novel therapeutics, (iii) optimize the clinical use of existing antiviral drugs, and (iv) enhance a quick response to emerging viral outbreaks.

## INTRODUCTION

Over the past century, many viral infectious diseases have caused substantial global morbidity and mortality (1, 2). The 1918 influenza pandemic, caused by the H1N1 influenza A virus, infected approximately 500 million people and resulted in at least 50 million deaths worldwide. Since its identification in 1983 (3), the human immunodeficiency virus (HIV) has claimed approximately 44.1 million lives, and 40.8 million people were living with HIV in 2024 (https://www.who.int/). The hepatitis B virus, first discovered in 1963 (4), causes liver damage and infects more than 250 million people, contributing to nearly 1.1 million deaths in 2022, according to the World Health Organization’s (WHO) Global Health Survey (https://www.who.int/). In the past two decades, major viral outbreaks have caused significant health and economic impacts, including the 2003 severe acute respiratory syndrome coronavirus (SARS-CoV) outbreak, the 2009 swine flu pandemic, the 2012 Middle East respiratory syndrome coronavirus (MERS-CoV) outbreak, the 2013–2016 Ebola virus epidemic in West Africa, the 2015–2016 Zika virus epidemic, the 2019 coronavirus disease (COVID-19) pandemic, the 2022–2023 mpox outbreak, and the 2024–2025 dengue outbreak. In the era of rapid global change, viral infections can spread rapidly due to factors such as increased international travel and trade, rapid urbanization, changing land-use patterns, and climate change (5). Except for smallpox, which has been eradicated, many viral infections continue to circulate in human populations, highlighting the urgent need for effective antiviral agents to control viral infectious diseases (6–14).

A dedicated open-access database of potent antiviral agents remains lacking. While many drug databases have been developed to cover medications for various human diseases (15–18), those focusing on antiviral agents—summarized in Table 1—have notable limitations. For instance, DrugBank 6.0 provides comprehensive drug-related information, including drug–drug interactions, drug–food interactions, and pharmaceutical data (19), but lacks clinical efficacy data and *in vitro* activity assay results (e.g., IC_50_, EC_50_, and CC_50_) that are critical for assessing antiviral potency. The Drug Repurposing Hub database offers compound-level annotations (e.g., compound name, clinical phase, mechanism of action, and protein target) and physical sample data (e.g., compound purity, expected mass, and PubChem ID) (20), but does not include antiviral activity data. The HIV Drug Resistance Database focuses on HIV drug resistance and *in vitro* data for HIV inhibitors in cell culture (21), while the Stanford Coronavirus Antiviral & Resistance Database covers SARS-CoV-2 drug resistance only (22). SIDER 4.1 database records adverse reactions for approved drugs (23), but it lacks clinical efficacy data. DrugVirus.info V2.0 offers basic information on approved antivirals and combinations (24), but lacks most quantitative potency data (e.g., IC_50_, EC_50_, and CC_50_). The DRAVP database contains antiviral peptides and proteins (25), but only a few peptides have been approved by the U.S. Food and Drug Administration (FDA) for antiviral use (26). VIROPENDIUM (https://www.antiviralintelistrat.com/) is a commercial database that compiles *in vitro* and *in vivo* data on antiviral drugs and vaccines; however, its access is restricted. The DrugCentral 2023 database lists approved drugs for human and veterinary diseases, along with indications, drug targets, adverse events, pharmacological classification, and off-patent status (27), but does not cover experimental inhibitors. Similarly, the Drug Information Database (https://www.phactmi.org/) offers label information for FDA-approved drugs, including dosage, contraindications, adverse reactions, drug interactions, storage, mechanism of action, and efficacy, but does not cover experimental antivirals.

**TABLE 1:** Comparison of AntiviralDB with other open-access drug databases

Database	Major content	Approved antivirals	Experimental antivirals	Antiviral targets	Clinical efficacy	Clinical guidelines	Drug screening protocols	Ref.
AntiviralDB	Expert-curated database of approved and experimental antivirals	Yes	Yes	Yes	Yes	Yes	Yes	This study
DrugBank 6.0	A database containing basic information on drugs and their targets	Yes	Yes	Yes	No	No	No	(19)
Drug Repurposing Hub	Curated database with drug annotations to support drug repurposing	No	Yes	Yes	No	No	No	(20)
HIV Drug Resistance Database	Database of HIV drugs and associated resistance profiles	Only HIV	No	Yes	No	No	No	(21)
Stanford Coronavirus Antiviral & Resistance Database	Curated database of SARS-CoV-2 variants and their susceptibility to monoclonal antibodies	Only COVID-19	Yes	Yes	No	No	No	(22)
SIDER 4.1	2015 database of marketed drugs and their adverse reactions	Yes	No	No	No	No	No	(23)
DrugVirus.info 2.0	Database of broad-spectrum antivirals and their combinations	Yes	Yes	No	No	No	No	(24)
DRAVP	Repository of antiviral peptides and proteins	No	Yes	Yes	No	No	No	(25)
DrugCentral 2023	This database includes drug indications, chemical structures, pharmacologic properties, and drug action	No	No	Yes	No	No	No	(27)
phactMI Drug Information Database	Medical information resources and expert consultation services	Yes	No	Yes	Yes^*a*^	No	No	(28)

^*a*^ Clinical efficacy data are currently available only for approved drugs, while such data remain unavailable for experimental agents undergoing phase 1, 2, or 3 clinical trials.

An open-access database dedicated to potent antiviral agents is critical for combating viral infectious diseases. However, existing databases literature (19–21, 23–27) is limited by (i) absence of clinical efficacy from randomized trials and quantitative *in vitro* data such as IC_50_, EC_50_, and CC_50_ across specific viral strains and cell lines; (ii) lack of official guidelines for antiviral drug use; and (iii) absence of standardized laboratory protocols for antiviral drug evaluation under biosafety conditions. To overcome existing limitations, we develop AntiviralDB—an open-source, expert-curated database that offers comprehensive information on antiviral agents to support effective management of viral infectious diseases in humans.

## MATERIALS AND METHODS

### Database development

The user interface was developed using HTML5, CSS 3.0, and JavaScript ES6, built on the Vue.js 2.6 framework with Element UI 2.11. For data storage, retrieval, and querying, MySQL 8.0 was used, with Redis 5.0 used to optimize database read speeds. Interactive data visualizations were implemented using ECharts 5.0 and a RESTful API, enabling users to dynamically explore the online database. To ensure fast and reliable access, a content delivery network was deployed to accelerate webpage-loading times. All connections are secured via HTTPS with an SSL certificate. AntiviralDB is hosted on the Alibaba Cloud platform, providing stable, high-performance computing and storage capabilities.

### Data sources for approved and experimental antiviral agents

The list of antiviral drugs approved before 1 August 2025 was retrieved from the records of relevant regulatory authorities, including the U.S. FDA, the European Medicines Agency (EMA), the National Medical Products Administration, and official government websites of the United Kingdom, Japan, Australia, Canada, and Switzerland. The latest versions of drug labels were downloaded to extract key information on dosage, approved indications, and clinical pharmacology. Clinical efficacy and safety data for approved antiviral drugs were obtained from phases 2, 3, or 4 randomized clinical trials by searching the clinical trial database at ClinicalTrials.gov. We utilized keywords such as “(antiviral agent) AND (virus name)” to search publications in PubMed, Google Scholar, and ChatGPT for data on antiviral agents with *in vitro* or *in vivo* activities against live viruses. Predicted inhibitors from computational models were excluded unless supported by experimental evidence. From the relevant publications, we collected data on drug parameters such as IC_50_, EC_50_, CC_50_, cell lines, virus strains, drug targets, and chemical structures. Protein data were retrieved from the Protein Data Bank (PDB). PyMOL V2.6 was used to generate 3D structural visualizations of drug targets. All sources were appropriately cited.

### Data sources of antiviral targets

Antiviral drug targets are categorized based on the structural and functional properties of viral proteins, including viral polymerases, proteases, integrases, structural proteins, and other viral proteins, as well as host proteins targeted by antiviral agents. For each viral target, information such as gene names, UniProt ID, PDB ID, functions in the viral life cycle, targeted drugs, and interacting proteins was compiled from PubMed, Google Scholar, UniProt, and the PDB.

### Data sources of wet-lab protocols and clinical guidelines

Standardized wet-lab protocols are crucial for antiviral drug discovery in biosafety laboratories. To this end, we compiled antiviral-related protocols from leading publications, including *Nature Methods*, *Nature Protocols*, *Nature Microbiology*, *Journal of Virology*, *PLoS Pathogens*, and *Antiviral Research*. Clinical guidelines were sourced from official organizations, such as the WHO, the U.S. National Institutes of Health, the International Antiviral Society–USA, the Infectious Diseases Society of America, the American Society for Microbiology, the American Academy of Pediatrics, the European AIDS Clinical Society, the British HIV Association, the National Institute for Health and Care Excellence, the European Association for the Study of the Liver, the American Association for the Study of Liver Diseases, the Chinese Medical Association, and the Centers for Disease Control and Prevention in the United States, Europe, and China.

## RESULTS

### The web interface and structure of AntiviralDB

**Fig 1:**
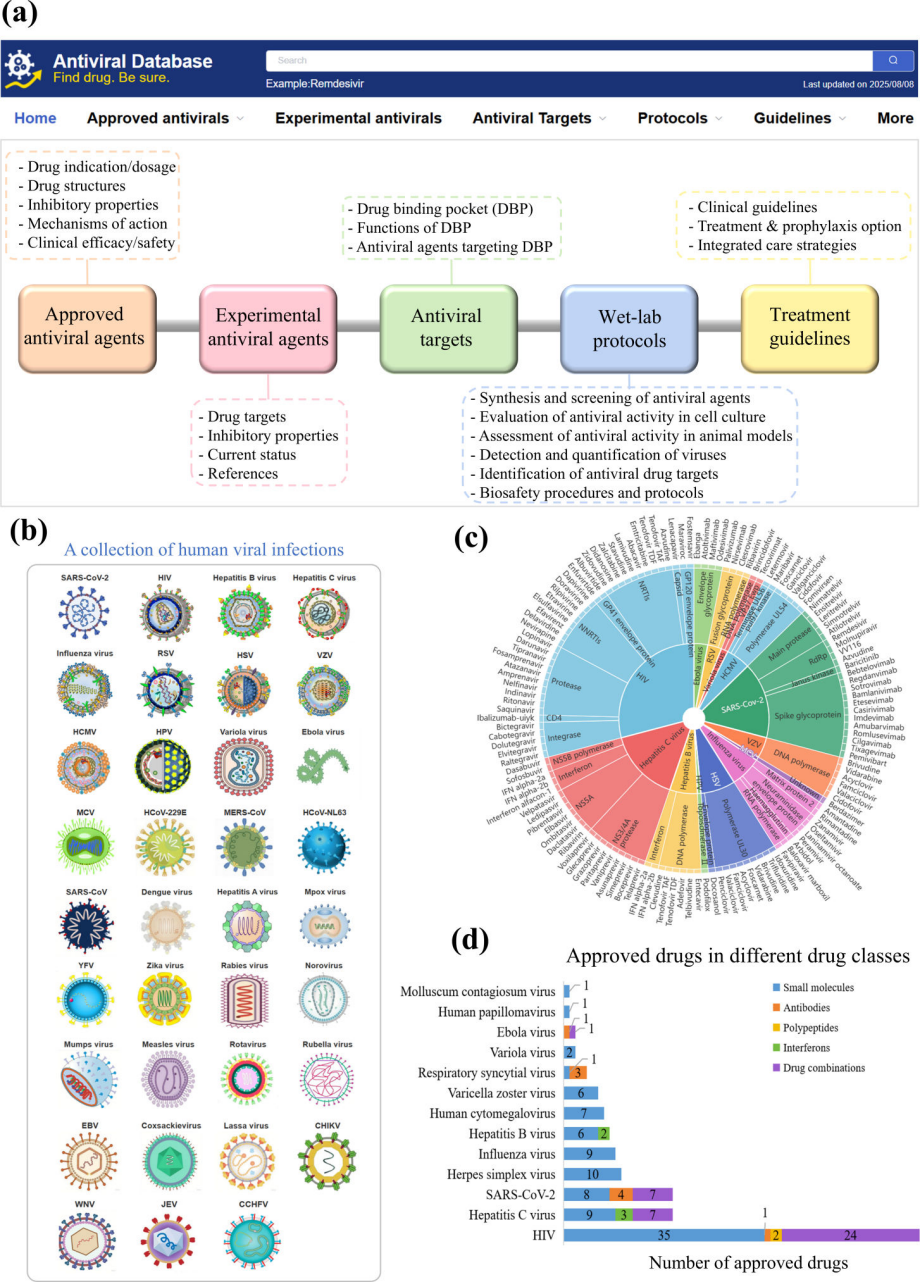
Data structure and functions of AntiviralDB. (**a**) The five main modules of the database and their respective contents. (**b**) Visualization of the human viruses covered in the database. (**c**) Interactive sunburst chart displayed on the homepage. (**d**) Number of approved drugs available for the management of different viral infections. RSV, respiratory syncytial virus; HSV, herpes simplex virus; VZV, varicella zoster virus; HCMV, human cytomegalovirus; HPV, human papillomavirus; MCV, molluscum contagiosum virus; YFV, yellow fever virus; EBV, Epstein-Barr virus; CHIKV, chikungunya virus; WNV, West Nile virus; JEV, Japanese encephalitis virus, CCHFV, Crimean–Congo hemorrhagic fever virus.

This study presents AntiviralDB, a comprehensive database designed to provide detailed information on antiviral agents for the management of human viral infections. As shown in Fig. 1a, AntiviralDB features a user-friendly interface that enables searches across five key areas of antiviral strategies: (i) approved antiviral drugs, including those authorized by the U.S. FDA, the EMA, and other regulatory agencies; (ii) investigational agents with potent antiviral activity in cell culture or animal models; (iii) antiviral drug-binding sites in viral proteins; (iv) validated laboratory protocols for virus culturing and antiviral discovery; and (v) clinical guidelines for the therapeutic use of antiviral drugs. The visualization tool (Fig. 1a) allows users to easily explore data on viruses, approved and experimental antiviral agents, and relevant guidelines and protocols. An interactive sunburst chart on the homepage provides quick access to common viral infections (Fig. 1b) and their approved antiviral treatments (Fig. 1c). As of 1 August 2025, a total of 98 antiviral agents and 39 combination therapies have been approved for the management of 13 human viral infections caused by HIV, HBV, HCV, SARS-CoV-2, human papillomavirus, Ebola virus, variola virus, respiratory syncytial virus, varicella zoster virus, human cytomegalovirus, herpes simplex virus, molluscum contagiosum virus, or influenza virus (Fig. 1d). These agents can be classified as small molecules (*N* = 84), antibodies (*N* = 9), peptide inhibitors (*N* = 2), interferons (*N* = 3), and combination regimens (*N* = 39) that comprise two or more antiviral drugs above.

### Antiviral agents and their targets

As of 1 August 2025, AntiviralDB includes 137 approved antiviral regimens and 1,386 experimental agents with laboratory-confirmed *in vitro* and/or *in vivo* activities against human viral infections. As summarized in Table 2, the database currently covers 35 human viruses from 17 viral families, including (i) *Herpesviridae*: hepatitis B virus, varicella zoster virus, human cytomegalovirus, herpes simplex virus, and Epstein–Barr virus; (ii) *Poxviridae*: variola virus, molluscum contagiosum virus, and mpox virus; (ii) *Papillomaviridae*: human papillomavirus; (iv) *Coronaviridae*: SARS-CoV-2, MERS-CoV, SARS-CoV-1, human coronavirus 229E, and human coronavirus NL63; (v) *Flaviviridae*: hepatitis C virus, dengue virus, yellow fever virus, Zika virus, West Nile virus, and Japanese encephalitis virus; (vi) *Picornaviridae*: hepatitis A virus and coxsackie virus; (vii) *Caliciviridae*: norovirus; (viii) *Togaviridae*: chikungunya virus; (ix) *Matonaviridae*: rubella virus; (x) *Orthomyxoviridae*: influenza virus; (xi) *Paramyxoviridae*: respiratory syncytial virus, mumps virus, and measles virus; (xii) *Rhabdoviridae*: rabies virus; (xiii) *Filoviridae*: Ebola virus; (xiv) *Arenaviridae*: Lassa virus; (xv) *Nairoviridae*: Crimean–Congo hemorrhagic fever virus; (xvi) *Reoviridae*: rotavirus; and (xvii) *Retroviridae*: HIV.

**TABLE 2:** Summary of 35 human viruses included in our database, AntiviralDB

Group	Family	Viruses covered by AntiviralDB	Experimental antiviral	Authorized antiviral drug^*a*^	Authorized vaccine^*a*^
dsDNA	*Herpesviridae*	Hepatitis B virus	Yes	Yes	Yes
		Varicella zoster virus	Yes	Yes	Yes
		Human cytomegalovirus	Yes	Yes	No
		Herpes simplex virus	Yes	Yes	No
		Epstein–Barr virus	Yes	No	No
	*Poxviridae*	Variola virus	Yes	Yes	Yes
		Molluscum contagiosum virus	Yes	Yes	No
		Mpox virus	Yes	No	Yes
	*Papillomaviridae*	Human papillomavirus	Yes	Yes	Yes
+ssRNA	*Coronaviridae*	SARS-CoV-2	Yes	Yes	Yes
		MERS-CoV	Yes	No	No
		SARS-CoV-1	Yes	No	No
		Human coronavirus 229E	Yes	No	No
		Human coronavirus NL63	Yes	No	No
	*Flaviviridae*	Hepatitis C virus	Yes	Yes	No
		Dengue virus	Yes	No	Yes
		Yellow fever virus	Yes	No	Yes
		Zika virus	Yes	No	No
		West Nile virus	Yes	No	No
		Japanese encephalitis virus	Yes	No	Yes
	*Picornaviridae*	Hepatitis A virus	Yes	No	Yes
		Coxsackie virus	Yes	No	No
	*Caliciviridae*	Norovirus	Yes	No	No
	*Togaviridae*	Chikungunya virus	Yes	No	Yes
	*Matonaviridae*	Rubella virus	Yes	No	Yes
−ssRNA	*Orthomyxoviridae*	Influenza virus	Yes	Yes	Yes
	*Paramyxoviridae*	Respiratory syncytial virus	Yes	Yes	Yes
		Mumps virus	Yes	No	Yes
		Measles virus	Yes	No	Yes
	*Rhabdoviridae*	Rabies virus	Yes	No	Yes
	*Filoviridae*	Ebola virus	Yes	Yes	Yes
	*Arenaviridae*	Lassa virus	Yes	No	Yes
	*Nairoviridae*	Crimean–Congo hemorrhagic fever virus	Yes	No	No
dsRNA	*Reoviridae*	Rotavirus	Yes	No	Yes
ssRNA	*Retroviridae*	HIV	Yes	Yes	No

^*a*^ Authorization information confirmed as of 1 August 2025.

**Fig 2:**
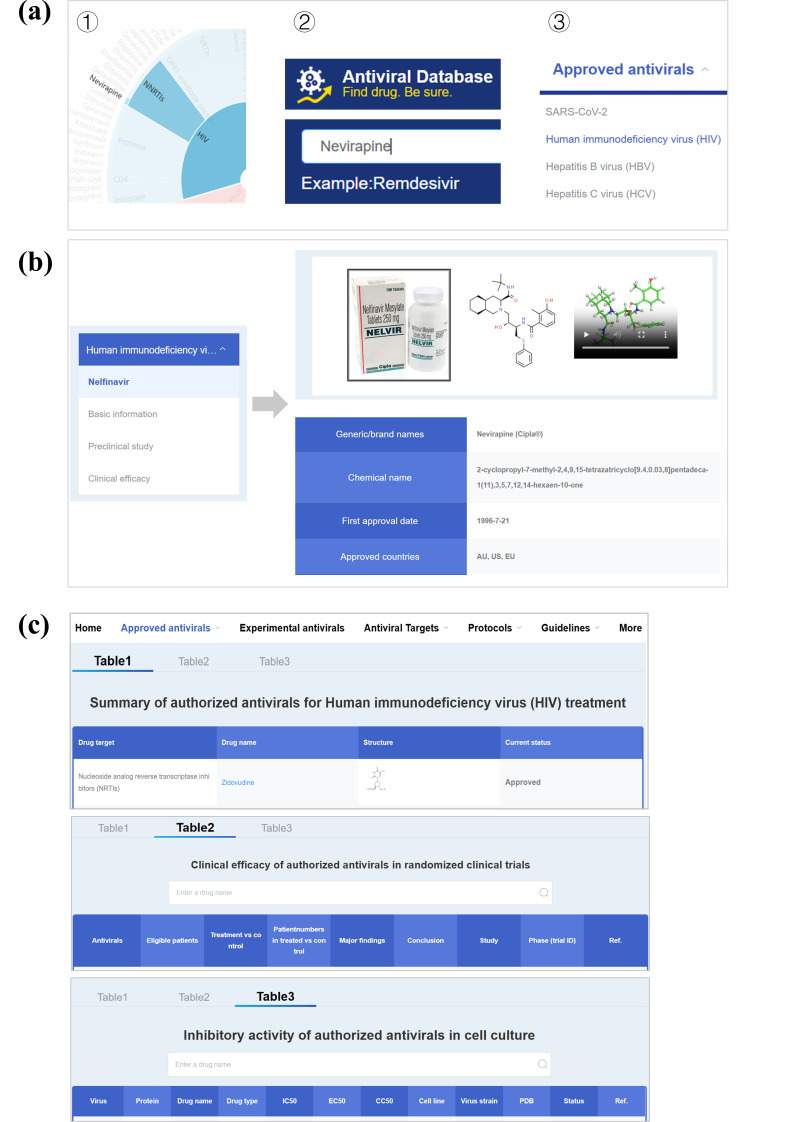
Antiviral information displayed in the database. (**a**) Three ways to access detailed drug information. (**b**) A list of antiviral drugs with their corresponding targets and chemical structures, each linked to a detailed profile page. (**c**) Three summary tables presenting the *in vitro* and *in vivo* activities of approved antiviral drugs.

Approved antiviral drugs can be accessed via the homepage sunburst chart or the search box. Virus-specific drug lists are available from the dropdown menu (Fig. 2a). For each drug, AntiviralDB provides information on clinical efficacy from clinical trials, adverse reactions, countries of approval, first approval date, chemical structure, mechanisms of action, and *in vitro* potency (IC_50_, EC_50_, and CC_50_) (Fig. 2b). As shown in Fig. 2c, the “Approved Antivirals” module summarizes drug targets, current status, clinical trial data regarding study design, efficacy and safety data, endpoints, primary and secondary outcomes, key findings, ClinicalTrials.gov identifiers (NCT numbers), and PubMed literature references.

**Fig 3:**
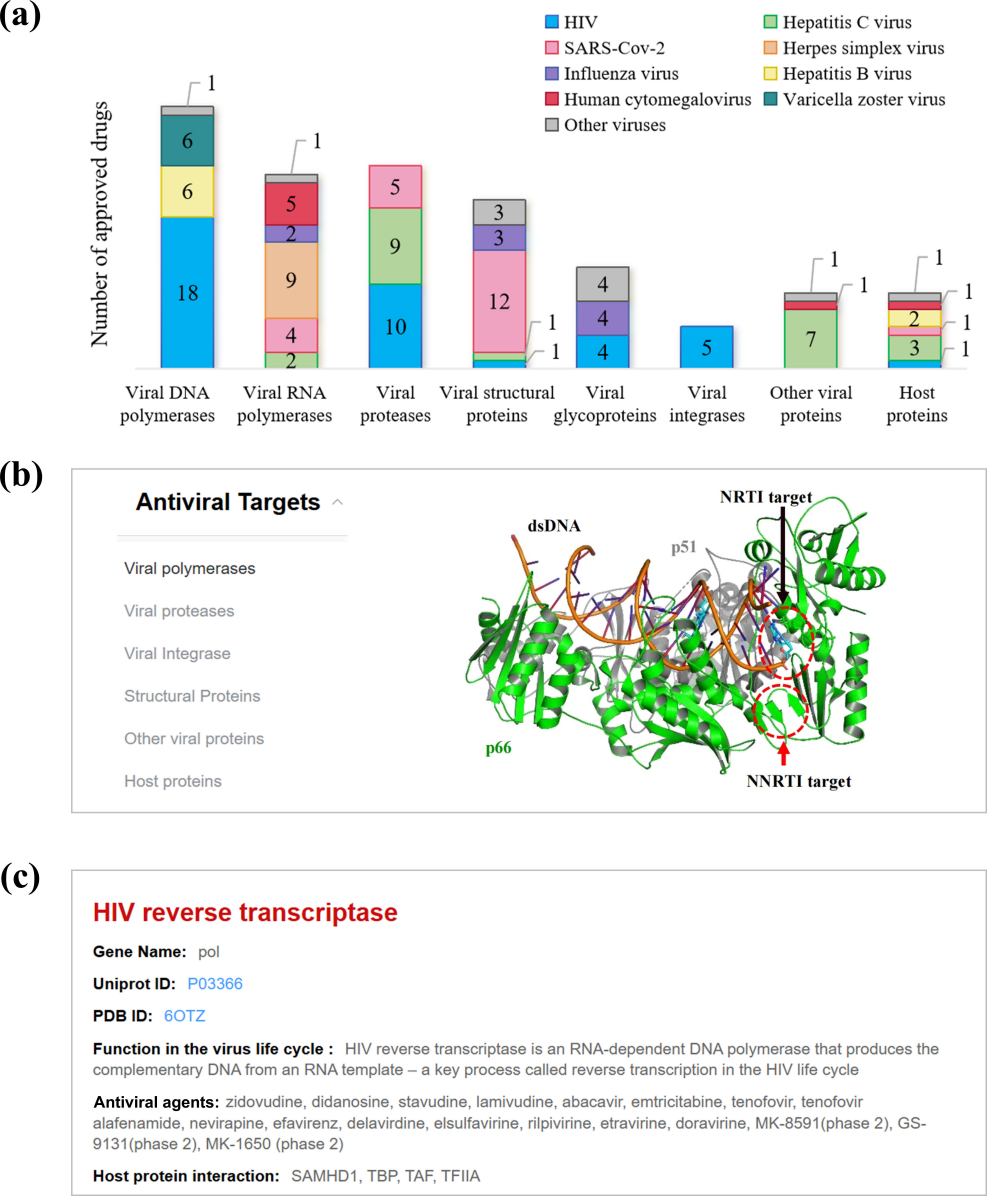
Antiviral targets and drug mechanisms in AntiviralDB. (**a**) Overview of antiviral targets among approved antiviral drugs. (**b**) Visualization of drug-binding pockets within protein structures. (**c**) Detailed description of specific target categories.

The success of mechanism-based drug discovery often depends on validating drug targets. To support this, the “Antiviral Targets” module highlights all potent drug targets. Based on the mechanisms of action of approved antiviral drugs, our database categorizes drug targets into six classes: viral polymerases, viral proteases, viral integrases, structural proteins, other viral proteins, and host proteins (Fig. 3a). By selecting a specific target in the “Antiviral Targets” module, users can find details on drug-binding pockets, approved drugs, protein functions, and protein–protein interactions (Fig. 3b). Hyperlinks to UniProt IDs and PDB IDs are provided for users to access additional resources (Fig. 3c). Importantly, 3D models of drug targets are freely available to support structural analysis and mechanism-based drug design.

### Antiviral activity of inhibitors in clinical trials and cell culture

**Fig 4:**
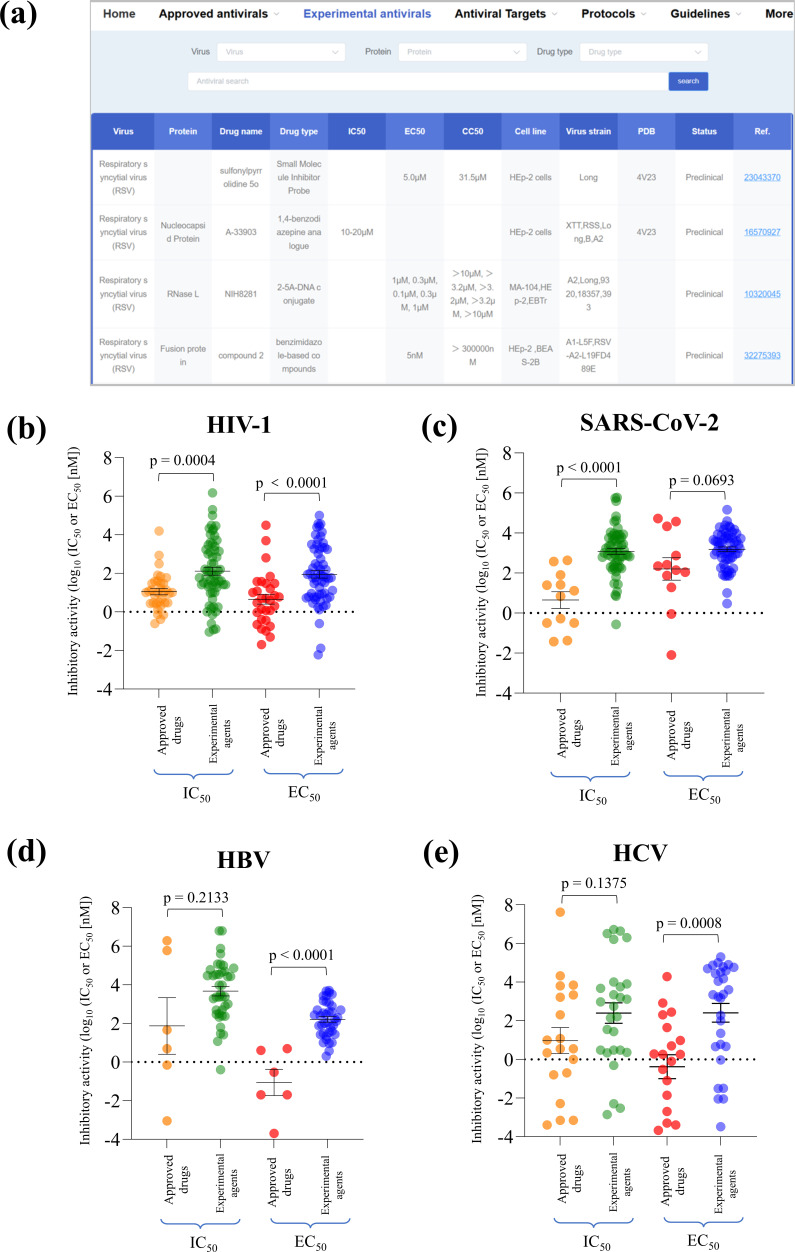
Inhibition activities of experimental inhibitors (**a**) and their comparisons with approved drugs. Comparisons of inhibitory activities against HIV-1 (**b**), SARS-CoV-2 (**c**), HBV (**d**), and HCV (**e**). Mann–Whitney tests were used to compare the differences in IC_50_ or EC_50_ values between two groups. HIV-1, human immunodeficiency virus type 1; HBV, hepatitis B virus; HCV, hepatitis C virus.

AntiviralDB compiles *in vitro* and *in vivo* activity data for approved (Fig. 2c) and experimental agents (Fig. 4a). For experimental inhibitors, which rarely have clinical trial data, the database summarizes *in vitro* results, including drug targets, IC_50_, EC_50_, and CC_50_ values, cell lines, virus strains, PDB entries, current status, and references. The antiviral activities of experimental inhibitors are compiled under the “Experimental Antivirals” module (Fig. 4a).

We compared the *in vitro* IC_50_ and EC_50_ values of approved and experimental agents against HIV-1 (Fig. 4b), SARS-CoV-2 (Fig. 4c), HBV (Fig. 4d), and HCV (Fig. 4e). In all four viral infections, the approved inhibitors consistently have lower potency data of IC_50_ and EC_50_ values, with statistically significant difference for HIV-1 (*P*-value < 0.05). However, some experimental compounds demonstrated better *in vitro* potency compared with approved drugs. For instance, nirmatrelvir-derivative 5 inhibited SARS-CoV-2 (IC_50_: 8 nM) and displayed potent broad-spectrum anti-coronaviral activity (29). In HIV-1 drug development, compound 1e displayed a strong inhibition (IC_50_: 90 pM) in an HIV-1 enzymatic inhibitory assay (30). 4′-Modified nucleoside analogs inhibited HBV with a low IC_50_ value of 0.4 nM (31). The compound 11, which incorporates a 4-silapiperidine group, demonstrated a strong anti-HCV activity (EC_50_: 0.33 pM) in an NS5A polymerase inhibition assay (32). Other potent experimental inhibitors have also been documented in AntiviralDB.

### Wet-lab protocols for antiviral drug discovery

**Fig 5:**
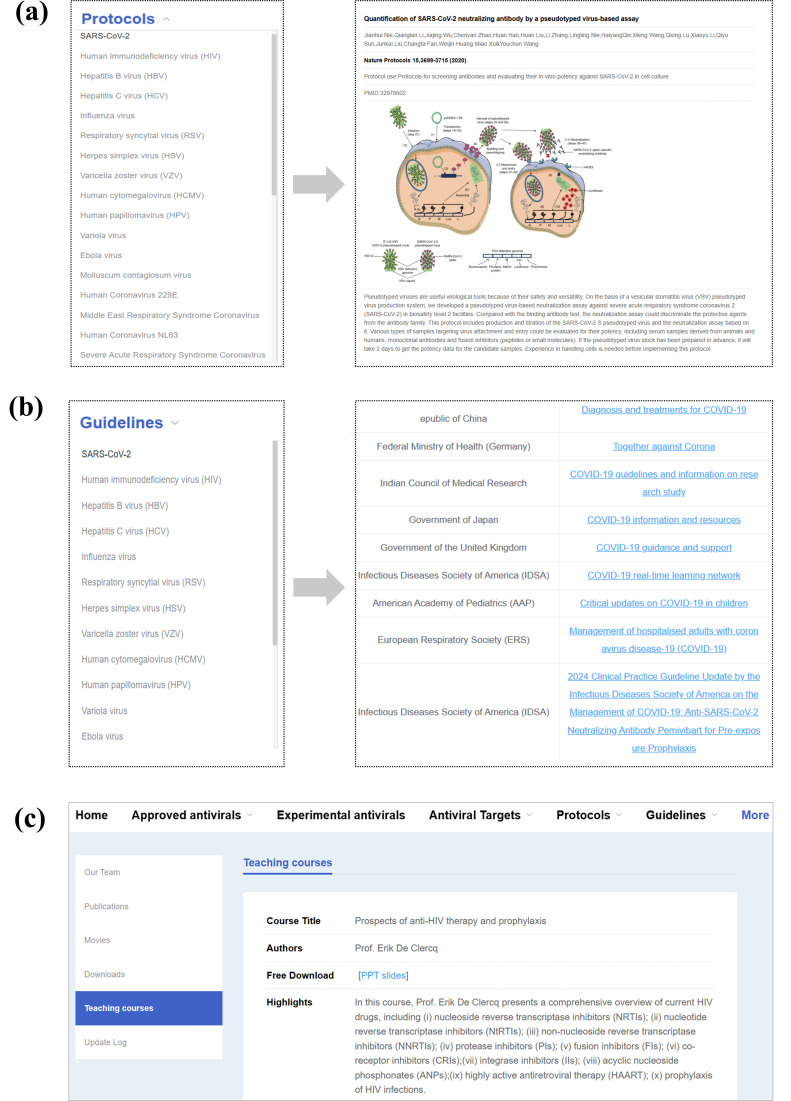
Protocol library and treatment guidelines in AntiviralDB. (**a**) Experimental protocols for antiviral drug discovery in the “Protocols” module. (**b**) Clinical treatment guidelines in the “Guidelines” module. (**c**) Features included in the “More” module.

Wet-lab protocols are critical for validating the potency of antiviral agents and ensuring reproducible results in cell culture and animal models. To support this, we have included a “Protocols” section in AntiviralDB, offering an extensive repository of standardized wet-lab protocols for antiviral drug development in biosafety laboratories. As shown in Fig. 5a, we collected protocols for various viral infections, including SARS-CoV-2, influenza virus, HIV, hepatitis B virus, hepatitis C virus, respiratory syncytial virus, herpes simplex virus, varicella zoster virus, human cytomegalovirus, human papillomavirus, Ebola virus, Mpox virus, MERS-CoV, SARS-CoV, dengue virus, hepatitis A virus, Zika virus, rabies virus, norovirus, measles virus, coxsackievirus, chikungunya virus, West Nile virus, and Japanese encephalitis virus. Protocols are organized into six categories: (i) synthesis and screening of antiviral agents; (ii) evaluation of antiviral activity in cell culture; (iii) assessment of antiviral activity in animal models; (iv) virus detection and quantification; (v) antiviral target identification; and (vi) biosafety procedures. Each protocol includes step-by-step instructions, equipment requirements, safety notes, and guidelines for replicating and validating *in vitro* and *in vivo* experiments.

### Clinical guidelines for the management of viral infections

Clinical guidelines are widely used by healthcare professionals and patients for managing viral infectious diseases. However, no existing database consolidates comprehensive international clinical guidelines for viral infections. To address this gap, the “Guidelines” module was added to include clinical treatment guidelines from global organizations and expert panels. The current version of our database covers 56 clinical guidelines for the management of 12 major viral infections. As shown in Fig. 5b, users can easily navigate these guidelines by specific viral infections and access information on pharmacological interventions, non-pharmacological treatments, and medical care strategies for both current and emerging infectious diseases. The guidelines are regularly updated to reflect the most recent recommendations.

### Additional resources

AntiviralDB is curated and maintained by a dedicated team of experts in virology, pharmacology, and clinical medicine. Under the “More” section (Fig. 5c), users can freely download all drug data sets, clinical guidelines, and protocols. To support scientific education and training, AntiviralDB also provides PowerPoint presentations that cover topics such as antiviral discovery and therapeutic strategies for viral infections. Contact details and team profiles are listed, along with references to peer-reviewed publications documenting database development and updates.

## DISCUSSION

The history of human infectious diseases over the past century highlights the critical role of antiviral agents in combating viral infections associated with high morbidity and mortality worldwide (33). In this context, this study presents AntiviralDB, an antiviral drug database that provides detailed information on approved and experimental antiviral inhibitors in combating 35 human viral infections. For approved antiviral regimens, the database provides data on chemical structures, mechanisms of action, drug targets, *in vitro* antiviral activities, clinical efficacy, safety profiles, and other key attributes. Experimental inhibitors are featured with quantitative antiviral data, such as IC_50_, EC_50_, and CC_50_ values, obtained from specific viral strains and cell lines. Beyond cataloging drugs, AntiviralDB also identifies the most widely targeted viral proteins, offers standardized laboratory protocols, compiles clinical guidelines, and provides educational resources. This integrated database has the potential to support computer-aided drug design (34) and high-throughput screening (35) for the discovery of novel antiviral therapies.

Distinct from other databases, AntiviralDB uniquely offers updated laboratory protocols to guide the design of both *in vitro* and *in vivo* antiviral experiments. Standardization is critical, as variations in experimental conditions can yield inconsistent results. For example, chloroquine was initially reported as a potent anti-SARS-CoV-2 inhibitor (EC_50_: 1.13 µM, CC_50_ > 100 µM in Vero E6 cells) (36). However, subsequent studies demonstrated that chloroquine failed to inhibit SARS-CoV-2 in human lung cells (37), in SARS-CoV-2-infected hamsters (38), and in humans (39). This discrepancy highlights the need for standardized laboratory protocols to ensure accurate identification of potent antiviral candidates. To address this, our database includes protocols for synthesizing antiviral compounds, generating virus-neutralizing antibodies, expressing viral proteins, and developing animal models for antiviral testing. These standardized protocols have the potential to standardize antiviral screening methods across laboratories worldwide.

Several virus-related databases have been reported in the literature, including NCBI Virus (40), the International Committee on Taxonomy of Viruses (41), ViralZone (42), Virus Particle Explorer DB v3.0 (43), Virus-Host DB (44), the Bacterial and Viral Bioinformatics Resource Center (45), the Multi-omics Portal of Virus Infection (46), the Reference Viral Database (47), the Viral Host Range Database (48), ViMIC (49), Integrated Microbial Genomes Virus V4 (50), and GISAID (51). These resources provide extensive information, such as viral taxonomy, genomic sequences, viral genomic structures, gene annotations, genetic variations, virus–host proteins, transmission routes, geographical distribution, and virus ecology or epidemiology (16). However, antiviral drug information is often incomplete or absent in literature (Table 1). Many databases focus on specific viruses or drug classes and lack detailed coverage of clinical efficacy, safety data, clinical guidelines, and experimental protocols. AntiviralDB addresses these gaps by not only incorporating essential drug information but also integrating critical clinical, experimental, and educational resources.

This study has several limitations. Although the current version covers 35 human viruses, future research should expand the database to include all viral infectious diseases and their antiviral targets. Of note, a large number of antiviral agents have been developed mostly for major viral infections, while self-limited or rare infectious diseases remain underrepresented. Incorporating predictive algorithms to estimate the potency of antiviral agents could further enhance the database, enabling repurposing of existing drugs for novel indications. Building on AntiviralDB, future research could prioritize the optimization and screening of small-molecule inhibitors with broad-spectrum activity against multiple viral infections.

### Conclusion

Over the past century, infectious diseases have posed a significant threat to global public health. This study presents AntiviralDB, an open-source, expert-curated database that integrates comprehensive antiviral data on antiviral agents, clinical guidelines, laboratory protocols, and educational materials. By supporting evidence-based management of human viral infections, AntiviralDB has the potential to accelerate the discovery of novel antiviral drugs, optimize existing therapeutic regimens, and strengthen preparedness for emerging viral threats.

## Data Availability

Our AntiviralDB data set is accessible at: https://www.antiviraldb.com/. The website is freely available to all users and does not require registration or login.
